# Biophysical Properties and Antiviral Activities of Measles Fusion Protein Derived Peptide Conjugated with 25-Hydroxycholesterol

**DOI:** 10.3390/molecules22111869

**Published:** 2017-10-31

**Authors:** Bárbara Gomes, Nuno C. Santos, Matteo Porotto

**Affiliations:** 1 Instituto de Medicina Molecular, Faculdade de Medicina, Universidade de Lisboa, Av. Prof. Egas Moniz, 1649-028 Lisbon, Portugal; bgomes@fm.ul.pt; 2 Center for Host-Pathogen Interaction, Columbia University Medical Center, 701 W. 168th St., New York, NY 10032, USA; 3 Department of Pediatrics, Columbia University Medical Center, 701 W. 168th St., New York, NY 10032, USA

**Keywords:** measles virus, fusion, peptide, 25-hydroxycholesterol

## Abstract

Measles virus (MV) infection is re-emerging, despite the availability of an effective vaccine. The mechanism of MV entry into a target cell relies on coordinated action between the MV hemagglutinin (H) receptor binding protein and the fusion envelope glycoprotein (F) which mediates fusion between the viral and cell membranes. Peptides derived from the *C*-terminal heptad repeat (HRC) of F can interfere with this process, blocking MV infection. As previously described, biophysical properties of HRC-derived peptides modulate their antiviral potency. In this work, we characterized a MV peptide fusion inhibitor conjugated to 25-hydroxycholesterol (25HC), a cholesterol derivative with intrinsic antiviral activity, and evaluated its interaction with membrane model systems and human blood cells. The peptide (MV–HC) has a 90% inhibitory concentration (IC_90_) several logs more advantageous than the equivalent peptide bearing a polyethylene glycol (PEG)-cholesterol moiety. In membrane interaction studies, MV–HC shows a preference for pure 1-palmitoyl-2-oleoyl-*sn*-glycero-3-phosphocholine (POPC) monolayers and membranes rich in sphingomyelin, and interacts less with POPC:cholesterol membranes. MV–HC tends to self-aggregate in aqueous solution, in a concentration-dependent manner. Our results suggest that increased membrane interaction dynamicity results from 25HC conjugation, with a concomitant increase in peptide antiviral efficacy.

## 1. Introduction

Measles virus (MV) is a human virus of the Paramyxoviridae family and *Morbillivirus* genus [1]. Despite the availability of a vaccine since 1963 and safe and effective immunization since the early 1980s, with a global drop in prevalence [2], measles is currently re-emerging and several recent outbreaks have occurred in developed countries. In the US, there were more than 600 measles cases in 2014, followed by the first measles-related death in the US in the last 12 years [3]. In 2017, outbreaks have also occurred in Romania and Italy [4]. Infections are mostly associated with vaccine refusal, but also occur in vaccinated persons exposed to this highly transmissible virus, and in the growing population of immunocompromised individuals [5]. The immune response elicited by vaccination varies widely within the population, the recommended two doses of vaccine are not a guarantee of an adequate protection [6,7], and immunocompromised people cannot be vaccinated with this live virus vaccine. The illness can be severe and lead to neurological sequelae, either immediately following infection or years later. Central nervous system (CNS) complications may occur soon after acute MV infection in the case of acute encephalomyelitis (AME), or years after infection, as a result of viral persistence in subacute sclerosing panencephalitis (SSPE). SSPE has recently been discovered to be more common than previously thought and may occur in up to one in 600 children infected under one year of age [8]. Despite presumably functional cell-mediated immunity and high antiviral antibody titers, immune control of CNS infection is not achieved in patients suffering from SSPE. The third form of MV-induced CNS disease—progressive infectious encephalitis or measles inclusion body encephalitis (MIBE)—occurs in immunosuppressed patients several months following MV infection [9,10,11]. There are no specific therapies for acute complications of MV or for persistent MV CNS infections [12,13,14,15,16].

Measles virus is an enveloped particle of approximately 200 nm, with the surface glycoproteins that mediate viral attachment and entry (hemagglutinin, H, and fusion, F) protruding from the cell-membrane derived lipid envelope, and the matrix proteins (M) lining the inner surface. The viral core is composed of the negative-sense RNA genome packaged with the nucleoprotein (N), large polymerase protein (L) and polymerase-associated protein (P) to form the replication complex. Entry of virus into a human cell and, presumably, cell-to-cell spread of virus generally requires interaction of the viral receptor binding protein (H) with host cellular receptor(s). Wild-type (wt) MV infection starts in the respiratory tract. Alveolar macrophages and dendritic cells are the primary targets [17,18,19,20]. Binding of the H protein to CD150 leads to initial infection. The first MV-infected cells then transmit the virus to bronchus-associated lymphoid tissues and/or draining lymph nodes, where the virus proliferates in B and T lymphocytes (that also express CD150), and viremia ensues [17,21]. The adherens junction protein PVRL4 (or nectin-4) [22,23,24,25,26,27] has been identified as a MV receptor on the basolateral surface of respiratory epithelial cells, associated with viral transmission at later stages of pathogenesis [21,26] as an exit receptor [28,29,30,31].

The H and F envelope glycoproteins work together to mediate virus attachment and entry into target cells. We refer to the H/F pairs of MV as the viral “fusion machinery” since these proteins, in the wt virus, act in concert. F is synthesized as a precursor (F_0_) that is cleaved within the cell to yield the pre-fusion F complex comprising three *C*-terminal F_1_ subunits linked covalently by disulfide bonds with three *N*-terminal F_2_ subunits. This trimeric F structure is kinetically trapped in a metastable conformation; the pre-fusion state is maintained only in the absence of activation by H or high temperature. Fusion activation occurs when H, upon engagement by a cell surface receptor (either CD150 or nectin-4, as discussed above), activates F [22,23,24,25,26,27]. After activation by H, the pre-fusion F undergoes a structural transition, extending and inserting its hydrophobic “fusion peptide” into the target membrane. During entry, F refolds into a “trimer of hairpin” post-fusion structure that brings together the *N*-terminal heptad repeat (HRN) and the *C*-terminal heptad repeat (HRC), and the viral and cell membranes fuse [32,33,34,35,36,37,38,39].

We and others have shown that peptides derived from the HRC region of the F_2_ ectodomain inhibit paramyxovirus entry with varying activity [35,40,41,42,43,44,45,46,47,48,49,50]. We have previously identified the antiviral activity of short peptides (36 amino-acid residues) corresponding to the HRC domain of paramyxovirus F.

We have shown that the peptides’ potency is determined by two factors: the strength of the peptide’s association with the corresponding HRN domain of the target fusion protein [49], and the kinetics of F activation by the virus’ attachment protein [51]. By targeting lipid-conjugated fusion inhibitory C-peptides to the plasma membrane, where fusion occurs, and by engineering peptides with increased HRN-HRC-peptide binding affinity, we have enhanced the antiviral potency of our prototype inhibitors by a factor of several logs [40,52,53,54]. Using lipid conjugation to increase the inhibitors’ half-life in vivo [53] and polyethylene glycol (PEG) linkers between the lipid moiety and the peptide, we further augmented potency and broad-spectrum activity [53]. For example, a specific HPIV3-derived, cholesterol-conjugated peptide is highly effective treatment for both the lethal encephalitis caused by the generally fatal paramyxovirus Nipah virus and for HPIV3 infection in vivo [53,55,56].

**Figure 1:**
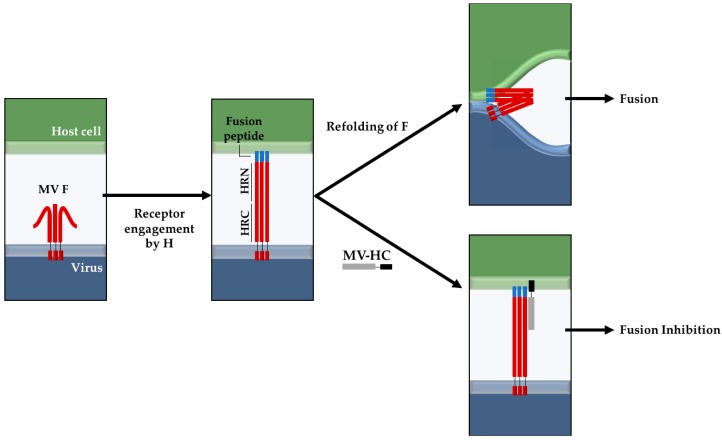
Proposed fusion inhibition mechanism of the MV–HC peptide. Peptides derived from the HRC domain of F can potently inhibit MV infection by binding to the HRN domain, after the fusion peptide inserts into the target cell, preventing the fusion between viral and cell membranes (adapted from [37,53]). For the sake of simplicity, the H protein is not represented in the diagram. The HRN and HRC domains, as well as the MV–HC peptide, are not represented up to scale relative to the full length of the protein or the thickness of the bilayer.

We have previously shown that specific biophysical properties of the HRC-derived peptides modulate their antiviral potency [56,57]. Specifically, we found that increasing the PEG linker length between the peptide and the cholesterol moiety led to a more dynamic interaction with model membranes, likely by decreasing the local hydrophobicity, and that this effect correlated with enhanced efficacy [56]. We therefore aimed to identify lipid modifications that would retain the anchoring property provided by cholesterol but increase the dynamicity of interaction with the membrane by being more hydrophilic. To this end, we evaluated a MV peptide fusion inhibitor derived from the HRC domain of MV F, conjugated to 25-hydroxycholesterol (25HC) without a PEG linker (Figure 1). 25HC is an oxysterol cholesterol derivative described in recent studies [58,59,60,61], more hydrophilic than cholesterol. To evaluate the potential of this conjugate, referred to as “MV–HC”, as an MV antiviral, we assessed its aggregation behavior and its interaction with biomembranes and human blood cells, since we have shown these properties to predictably correlate with antiviral effects [57].

## 2. Results

### 2.1. Interaction with Small Unilamellar Vesicles

**Figure 2:**
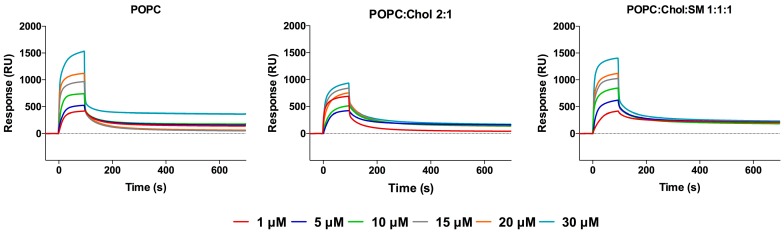
Evaluation of MV–HC peptide interaction with membranes. SPR sensorgrams obtained for MV–HC in the presence of POPC, POPC:Chol = 2:1 or POPC:Chol:SM = 1:1:1 membranes (1 mM). A range of peptide concentrations (1–30 µM) was injected over the deposited vesicles.

Kinetic profiles (sensorgrams) of peptide binding to small unilamellar vesicles (SUV) surfaces were acquired using surface plasmon resonance (SPR), which allows real-time detection of bound molecules on a SUV-covered surface [62] (Figure 2). Each sensorgram is composed of sequential binding and unbinding phases, corresponding to the sample injection over the lipid surface, followed by the removal of bound sample molecules through solvent flow. Three different membrane compositions were tested for their peptide binding, to mimic the liquid disordered domains containing 1-palmitoyl-2-oleoyl-*sn*-glycero-3-phosphocholine (POPC) and liquid ordered domains from mammalian cell membranes, with POPC and cholesterol (Chol), as well as lipid rafts containing sphingomyelin (SM) (POPC:Chol:SM), since it has been suggested that MV virions emerge from cholesterol rich-domains, such as lipid rafts [63,64], and may retain this lipid composition in the viral envelope. SUV stability and size distribution were evaluated by dynamic light scattering (DLS), using a Malvern Zetasizer NanoZS (Malvern Instruments, Malvern, UK), guaranteeing a homogeneous vesicle size distribution regardless of lipid composition.

**Figure 3:**
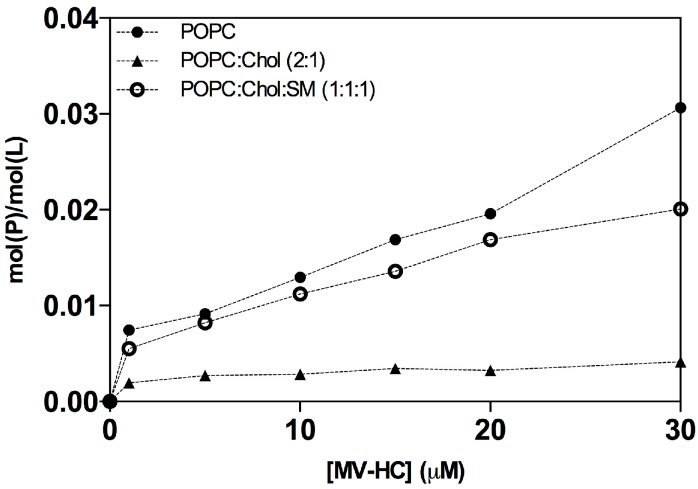
Average peptide-to-lipid ratios (mol(P)/mol(L)). The ratios were calculated using the sensorgrams’ maximum binding response, as a function of peptide concentration, for its interaction with deposited POPC (solid circles), POPC:Chol (2:1) (solid triangle) and POPC:Chol:SM (1:1:1) (open circle) SUV. Peptide binding and SUV capture response values were retrieved from individual sensorgrams at specific reporting time points. Molar ratio values were calculated based on the relationship 1 RU to 1 pg/mm^2^ of bound peptide or lipid [65].

MV–HC sensorgrams showed an interaction for all membranes tested (Figure 2). The highest level of binding and subsequent unbinding was observed for POPC vesicles, indicating a higher affinity of the peptide for disordered domains and dynamic unbinding. The addition of cholesterol decreases this interaction and unbinding, but the dynamicity increases in the presence of SM. The interaction between the conjugated peptide and the lipids is transient: the peptide binds to the lipid surface through a fast process, slowing down significantly until the end of peptide injection. Almost all peptide is removed during the unbinding phase, following a similar pattern, with a rapidly decreasing signal. Based on the maximum peptide binding and SUV deposition response, the respective average peptide-to-lipid molar ratios were calculated and plotted as a function of the injected peptide concentration (Figure 3).

The data in Figure 3 show a general increase in binding concomitant with increasing proportions of peptide. As seen in the sensorgrams, there is a significant interaction between the peptide and the POPC membranes. A weaker interaction was obtained for the POPC:Chol = 2:1 mixture, possibly explaining the dynamic behavior seen with this conjugate. In the presence of SM, the peptide’s interaction increases, suggesting that membranes bearing both cholesterol and SM interact well with the peptide conjugate, with very fast binding and unbinding processes.

### 2.2. Interaction with Blood Cells

**Figure 4:**
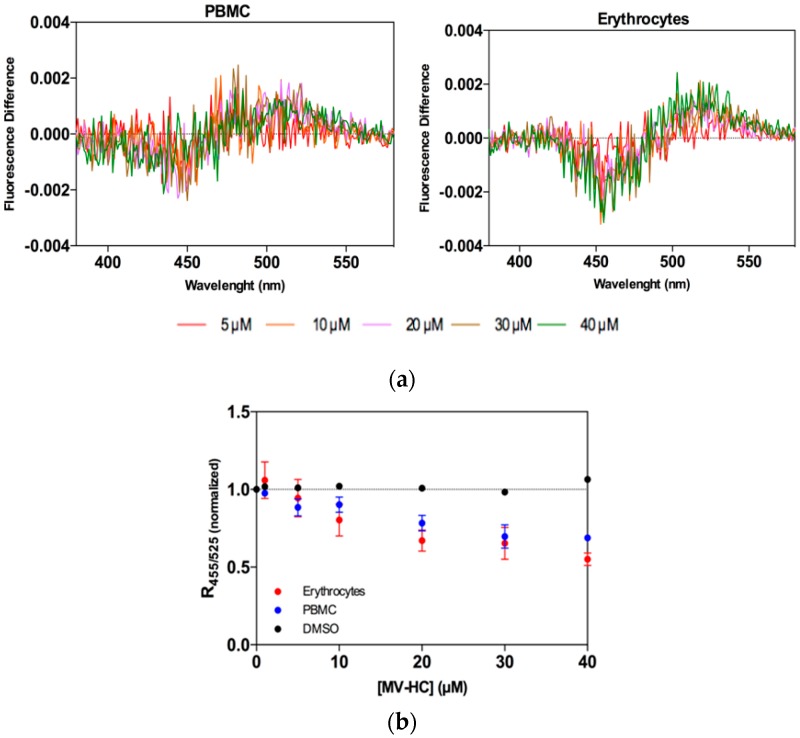
Peptide affinity towards erythrocytes and PBMC assessed by di-8-ANEPPS fluorescence: (**a**) Differential spectra of di-8-ANEPPS bound to cells in the presence of a range of concentrations of MV–HC. Spectra were obtained by subtracting the excitation spectrum (normalized to the integrated areas) of labeled cells in the absence of peptide from those in its presence (different concentrations); (**b**) Binding profiles of MV–HC to erythrocytes and PBMC, obtained by plotting the di-8-ANEPPS excitation ratio, R_455/525_, normalized to the initial value, as a function of the peptide concentration.

Erythrocytes and peripheral blood mononuclear cells (PBMC) were selected as cell models for assessing peptide–cell membrane affinity, and the lipophilic fluorescent probe di-8-ANEPPS was used as an indirect reporter of peptide–lipid interaction. Erythrocytes were chosen for study as a potential carrier for antiviral peptides during measles viremia (virus circulating in the blood stream) and PBMC are important since MV targets these cells after the initial respiratory phase. MV–HC was incubated with the two cell types at a range of concentrations for 1 h. This peptide interacts with both types of cell, as shown by the dipole potential decrease in the presence of the peptide (Figure 4a). As a quantitative measure of the interaction of MV–HC with membranes, the intensity ratio was calculated for each peptide concentration. A decrease in ratio values indicates an increase in peptide interaction, concomitant with the increasing concentrations of peptide (Figure 4b). The affinity is higher when compared to sifuvirtide, very similar to that obtained for enfuvirtide, and slightly lower than that for T-1249, three viral fusion inhibitory peptides analyzed in previous studies that target human immunodeficiency virus (HIV) [66,67]. Compared with other viral fusion inhibitory peptides that bear a lipid moiety [68,69], the results in Figure 4 show a lower interaction between MV–HC and the two cell types.

### 2.3. Aggregation Studies

**Figure 5:**
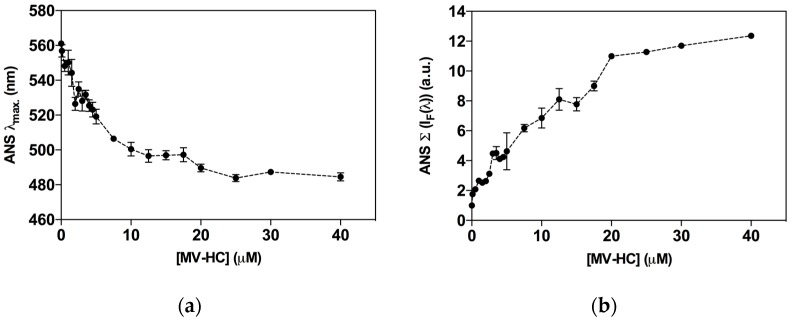
Aggregation of MV–HC evaluated by ANS fluorescence properties. The fluorescence emission maximum wavelength (λ_max_) (**a**); and the fluorescence intensity spectrum integral (Σ [I_F_(λ)]) (**b**) were plotted as a function of MV–HC concentration.

Self-association of peptide–lipid conjugates into higher order structures is modulated by amino acid sequence of the peptide and the nature of the lipid moiety [56,57]. The aggregation profile may directly influence the peptide’s behavior in the physiological environment and influence antiviral activity. The fluorescent probe 8-anilinonaphthalene-1-sulfonic acid (ANS) was used to assess the peptide’s propensity to self-assemble in aqueous solution. Interaction of ANS with hydrophobic sites causes an increase in its intrinsic fluorescence and a blue shift in the fluorescence emission maximum that correlates with peptide aggregation [70]. In the presence of ANS, increasing concentrations of MV–HC induced a blue shift in fluorescence emission, indicating the formation of aggregates (Figure 5a). A spectral maxima shift of approximately 80 nm was observed and was constant for concentrations above 20 μM. At the higher concentrations tested, ANS was mainly associated with the peptide aggregates. ANS fluorescence intensity as a function of the peptide concentration was also calculated (Figure 5b). At the highest concentration of peptide tested (40 μM), the fluorescence intensity recorded was about 12-fold higher than the control obtained in the absence of peptide, which suggests that MV–HC aggregates extensively in solution.

**Figure 6:**
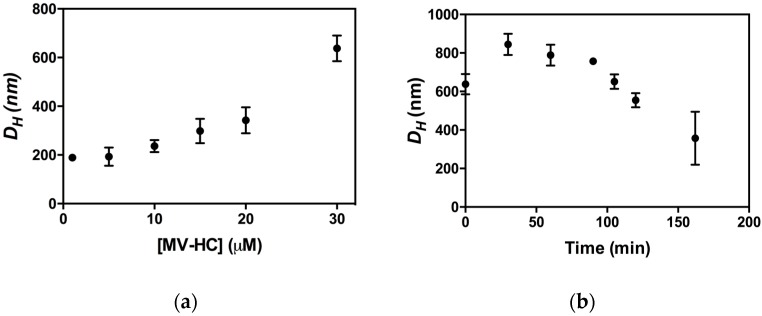
Aggregation of MV–HC evaluated by DLS: (**a**) average hydrodynamic diameter (D_H_) of the aggregates obtained at different MV–HC concentrations (time = 0 min); and (**b**) D_H_ values of MV–HC 30 μM followed over time.

The peptide aggregates were studied by DLS to determine their dimensions, presented here as hydrodynamic diameter (D_H_) [71]. The size of the aggregates is shown as a function of peptide concentration (Figure 6a) and time (Figure 6b). MV–HC aggregates even at the lowest concentration tested (1 μM). The average size of the aggregates depends on peptide concentration, with larger D_H_ values observed at higher concentrations. This behavior was also observed for dimers of HRC-derived peptides bearing a lipid moiety [57]. Nevertheless, the aggregates formed by MV–HC have a higher average hydrodynamic diameter. The peptide aggregation profile at 30 μM over time (Figure 6b) starts with an increase in average diameter of the self-assembled particles, followed by a progressive decrease, which may indicate particle precipitation.

### 2.4. Lipid-Conjugated Inhibitory Peptides Inhibit Cell-to-Cell Fusion

We have previously identified a strong correlation between efficiency of inhibiting cell-to-cell fusion in vitro, and in vivo potency [72]. We have attributed the enhanced in vivo efficacy of specific peptides to their membrane insertion properties identified in vitro [52]. Here we describe the biophysical properties of 25HC conjugated peptide and we found different membrane interaction (i.e., a significantly more dynamic and improved interaction, as discussed above) than the previously described HRC2 (see Table 1) [57].

A similar dynamic interaction was observed for a HPIV3-derived peptide with a monodisperse PEG24 [56]. To determine whether the increased dynamic interaction affects in vitro potency, we directly measured fusion inhibition. To assess fusion, the fusion inhibitory peptides were assessed using a semi-quantitative β-galactosidase (β-gal) complementation assay [74]. This assay measures the fusion of cells that express viral envelope glycoproteins (MV IC323 H/F) with cells that express the MV receptor SLAM-4 (Table 2).

Table 2 shows the peptide concentrations that blocked 50% (IC_50_) and 90% (IC_90_) of fusion. As previously shown, the addition of a lipophilic moiety increased the antiviral potency of the HRC peptides, compared to the untagged peptides [57]. When we compared the IC_50_ of MV–HC vs. the previously described HRC2 (conjugated with PEG–Chol), the difference in potency was not statistically significant; however, the IC_90_ of MV–HC was several logs better than the HRC2. These data are in agreement with our previous findings that correlated peptide efficacy to membrane dynamic interactions [56].

## 3. Discussion

The MV–HC peptide conjugate comprises two antiviral moieties: a fusion inhibitory peptide based on the *C*-terminal heptad repeat of the MV F protein, and 25-hydroxycholesterol, a cholesterol derivative with activity against a variety of enveloped viruses [59]. MV–HC preserves the membrane interaction property conferred by cholesterol conjugation and increases the dynamicity of the kinetics of interaction in membranes representative of cell types relevant to MV infection [57] We hypothesize that the presence of 25HC influences the biophysical behavior of the peptide complex, which may be important not only for in vitro activity but also for in vivo antiviral properties [68,69].

In this study, we examined interaction of the MV–HC peptide with model membranes and with human blood cells. The peptide showed a preference for vesicles of POPC and a lower affinity for a mixture of POPC and cholesterol (2:1). With the ternary mixture—POPC:Chol:SM (1:1:1)—the peptide showed an interaction similar to that observed for pure POPC, with a dynamic kinetics of binding and rapid unbinding. In order to test peptide–cell membrane affinity, we used the probe di-8-ANEPPS to evaluate the interaction of the peptide with blood cell membranes. MV–HC interacts both with erythrocytes and PBMC, with an affinity comparable to that previously determined for enfuvirtide, an HIV fusion inhibitor in clinical use [66].

The aggregation profile of MV–HC was assessed by two different approaches. The fluorescence spectroscopy-based ANS method relies on the differences of the fluorescence spectra of this probe in the absence and presence of a range of peptide concentrations, promoted by the interaction of ANS with hydrophobic sites [75]. An increase in peptide concentration was followed by an increase in fluorescence intensity, as well as by a blue shift on fluorescence emission, both indicating the formation of peptide aggregates. DLS analysis of the aggregates showed that the peptide starts to aggregate at the lowest concentrations tested, and the size of the self-assembled particles depends on the peptide concentration. At the highest concentration (30 μM), the average diameter of the particles decreased with time, which may suggest that the largest particles precipitate (becoming undetectable on the dynamic light scattering measurements), or reorganize into small aggregates. Such aggregation profile may eventually influence peptide insertion into the membrane and promote structural and dynamical changes in the lipid bilayer. Thus, the implication of peptide aggregation for peptide–lipid interaction is important to be clarified. Changes on the physical properties of the membranes, including membrane curvature and thickness, may influence the peptide’s mechanism of action. Note however that in vivo, peptides will be dispersed in a membrane-rich environment and likely in contact with their target membranes.

Our results suggest that 25HC conjugation increases the interaction between the peptide and the membrane to a level similar to that observed for HRC-derived peptides bearing a PEG24 linker between the peptide and the cholesterol conjugate. The PEG24 linker, while bestowing increased antiviral potency, also conferred sensitivity to proteolytic degradation [56]. Even with equivalent antiviral potency for the MV–HC conjugate compared with PEG24-linked MV–HRC peptides, a decrease in sensitivity to proteolytic degradation would offer a significant advantage.

We previously showed that cholesterol-conjugated HRC-derived antiviral peptides distribute into the brain and prevent otherwise lethal measles encephalitis [54,57,73]. Most drugs translocate the blood–brain barrier (BBB) by transmembrane fusion [76]. However, the small size of a molecule or supramolecular assembly is not a guarantee of BBB crossing. In fact, just 2% of small molecules with a molecular mass under 500 Da can cross the brain capillary endothelium by simple diffusion, lipophilicity being one of the keys for success [77,78]. Nevertheless, a balance is needed regarding lipid solubility. While increased lipophilicity can favor the transport rate across BBB, it also can lower the amount of drug that reaches the cells behind the BBB, since it decreases their partition into the aqueous environment of the brain’s interstitial fluid and increases their entrapment in the membranes [79]. An increase in lipid solubility promotes the uptake of the compound by the peripheral tissues, which can lead to lower concentrations of the compound available in the blood [76,80]. Thus, it will be of great interest to determine whether the altered hydrophobicity of MV–HC conjugated 25HC enhances brain distribution.

## 4. Material and Methods

### 4.1. Reagents

MV–HC (Ac–PPISLERLDVGTNLGNAIAKLEDAKELLESSDQILR–GSGSG–C–25HC) was obtained by custom synthesis (American Peptide Company, Sunnyvale, CA, USA). HEPES, NaCl and DMSO were from Merck (Darmstadt, Germany). POPC (1-palmitoyl-2-oleoyl-*sn*-glycero-3-phosphocholine) and SM (egg sphingomyelin), were purchased from Avanti Polar Lipids (Alabaster, AL, USA), while cholesterol (Chol) and Pluronic F-127 were from Sigma (St. Louis, MO, USA). Biacore sensor chip regeneration reagents, namely, 3-[(3-cholamidopropyl)dimethylammonio]-1-propanesulfonate (CHAPS) and methanol, were also purchased from Sigma. The fluorescent probes 8-anilino-1-naphthalenesulfonic acid (ANS) and 4-(2-[6-(dioctylamino)-2-naphthalenyl]ethenyl)-1-(3-sulfopropyl)pyridinium inner salt (di-8-ANEPPS) were from Merck (Darmstadt, Germany) and Invitrogen (Carlsbad, CA, USA), respectively. Lymphoprep was from Axis-Shield (Oslo, Norway).

The genes of MV IC323 wt H and wt F were codon optimized, synthesized and subcloned into the mammalian expression vector pCAGGS. The construct for SLAM was commercially acquired.

### 4.2. Sample Preparation

The lyophilized peptide was solubilized in DMSO up to 40 mg/mL, sonicated in a water bath for 5 min, and stored at −20 °C. Working peptide samples at defined final concentrations were solubilized from stock solutions in 10 mM HEPES 150 mM NaCl buffer, pH 7.4. The final DMSO content was maintained at 2% (*v*/*v*) in all experiments. Working peptide samples were sonicated in an ultrasonic bath for 2 to 5 min before use.

Small unilamellar vesicles (SUV) were prepared as previously described [81]. The lipid mixture was first solubilized in chloroform in a round-bottom flask. The solvent was evaporated under nitrogen flow until a thin lipid film was formed on the flask wall. The lipid film was further dried under vacuum overnight. A multilamellar vesicles (MLV) suspension was obtained after rehydration with the sample buffer and a series of 10 freeze–thaw cycles. The MLV suspension was extruded through a 50 nm-pore-size Nuclepore polycarbonate membrane purchased from Whatman/GE Healthcare (Maidstone, UK) using a LiposoFast-Basic plus Stabilizer setup from Avestin (Mannheim, Germany). This allowed the reorganization of MLVs into SUV. POPC, POPC:Chol (2:1), and POPC:Chol:SM (1:1:1) mixtures were prepared.

### 4.3. Surface Plasmon Resonance

For surface plasmon resonance (SPR) measurements, 10 mM HEPES 150 mM NaCl, pH 7.4, with 2% (*v*/*v*) DMSO was used as running buffer to match the peptide and lipid sample buffer composition. Experiments were performed in a GE Healthcare Biacore X100 (GE Healthcare Life Sciences, Buckinghamshire, UK). The system was primed at least three times with running buffer before starting an experiment. L1 sensor chips, designed for lipid binding assays, were used in all experiments. Immediately before each assay, the sensor chip surface was rinsed with three injections of 20 mM CHAPS. To prepare the lipid surface, a 1 mM POPC, POPC:Chol (2:1) or POPC:Chol:SM (1:1:1) SUV sample was injected over the sensor chip at a 2 µL/min flow speed for 2400 s (40 min). Loosely bound vesicles were removed with a 36 s injection of 10 mM NaOH, at 50 µL/min. MV–HC samples, with concentrations between 1 and 30 µM, were injected over the deposited lipid surface at a 5 µL/min flow speed during 200 s. Each sample was allowed a 600 s (10 min) unbinding time after injection stopped. After each run, the sensor chip surface was regenerated with sequential injections of 20 mM CHAPS (5 µL/min for 60 s), 0.5% (*w*/*v*) SDS (5 µL/min for 60 s), 10 mM NaOH with 20% (v/v) methanol (50 µL/min for 36 s), and 10 mM NaOH (50 µL/min for 36 s). Response values were monitored to ensure effective surface regeneration. To analyze the SPR membrane interaction data, the SUV deposition on the sensor chip surface and the response values for peptide binding to lipid were converted into units of moles per area and used to calculate the respective peptide-to-lipid molar ratio. To perform these calculations, we considered 1 response unit (RU) to be approximately 1 pg/mm^2^ of peptide or lipid, as previously described [82].

### 4.4. Membrane Dipole Potential Assessment with Di-8-ANEPPS

Human blood samples were obtained from healthy volunteers, with their previous written informed consent, at Instituto Português do Sangue (Lisbon, Portugal), as approved by the joint Ethics Committee of Faculdade de Medicina da Universidade de Lisboa and Santa Maria Hospital. Isolation of erythrocytes and peripheral blood mononuclear cells (PBMC), as well as the labeling of these cells with di-8-ANEPPS, were performed as previously described [66,67]. In the case of erythrocyte isolation, blood samples were centrifuged at 1200× *g* during 10 min to remove plasma and the buffy-coat. Erythrocytes were washed twice with the working buffer and then incubated at a 1% suspension in buffer supplemented with 0.05% (*m*/*v*) Pluronic F-127 (Sigma) and di-8-ANEPPS 10 µM. PBMC were isolated by density gradient using Lymphoprep and counted in a MOXI Z Mini Automated Cell Counter (Orflo Technologies, Ketchum, ID, USA). They were then incubated at a density of 3000 cells/mL in Pluronic-supplemented buffer with di-8-ANEPPS. Cells were incubated with the fluorescent probe during 1 h at room temperature, with gentle agitation, protected from light. Unbound probe was washed with Pluronic-free buffer on two centrifugation cycles.

Excitation spectra and the ratio of intensities at the excitation wavelengths of 455 and 525 nm (R = I_455_/I_525_) were obtained with emission set at 670 nm to avoid membrane fluidity-related artifacts [83,84]. Excitation and emission slits for these measurements were set to 5 and 10 nm, respectively.

### 4.5. ANS Fluorescence Studies

For the peptide aggregation studies, a 12.8 μM 8-anilinonaphthalene-1-sulfonic acid (ANS) solution was titrated with the MV–HC peptide up to final concentrations between 0 and 40 μM. For each sample, fluorescence emission spectra were collected between 400 and 600 nm (excitation wavelength = 369 nm) in a Varian Cary Eclipse fluorescence spectrophotometer (Mulgrave, Australia). Excitation and emission slits were 5 and 10 nm, respectively. A 10 min incubation time was allowed before each measurement. The fluorescence emission intensity values were corrected for dilution and background noise. The maximum emission wavelength, λ_max_, and the spectrum integral, [*I*_F_(λ)], were determined for each spectrum.

### 4.6. Dynamic Light Scattering

For dynamic light scattering (DLS), MV–HC peptide solutions with concentrations ranging from 1 to 30 μM were prepared as described above and incubated at 25 °C for 5 min before each measurement of particle size. Measurements were performed on a Malvern Zetasizer Nano ZS (Malvern Instruments, Malvern, UK) and consisted of 15 individual runs, each corresponding to an averaged autocorrelation curve obtained from at least 12 repeated sample scans. The diffusion coefficient (D) values were calculated from autocorrelation curves using a CONTIN-based method [85]. D values were used to determine averaged hydrodynamic diameter (D_H_) profiles through the Stokes–Einstein equation [86].

### 4.7. Cells

293T cells (human kidney epithelial cells) were grown in Dulbecco’s modified Eagle’s medium (DMEM) (GIBCO; Invitrogen, Carlsbad, CA, USA) supplemented with 10% fetal bovine serum and antibiotics, in 5% CO_2_. The Vero-SLAM culture medium was supplemented with geneticin.

### 4.8. Transient Expression of H and F Genes

Transfections were performed in 293T cells according to the Lipofectamine 2000 manufacturer’s protocols (Invitrogen, Carlsbad, CA, USA).

### 4.9. β-Galactosidase Complementation-Based Fusion Assay

This assay was performed as described previously [74,87]. Briefly, 293T cells transiently transfected with SLAM and the omega reporter subunit were incubated with cells coexpressing viral glycoproteins (MV H and MV F) and the alpha reporter subunit.

### 4.10. Data Analysis

The fitting of the experimental data with the equations mentioned in this article was done by non-linear regression using Prism 5 (GraphPad Software, La Jolla, CA, USA). Error bars on data presentation represent the standard error of mean (SEM).

## Figures and Tables

**Table 1:** Sequences and modifications of MV HRC derived peptides.

Peptide	Sequences and Modifications
**MV HRC1 ^a^**	Ac–PPISLERLDVGTNLGNAIAKLEDAKELLESSDQILR-GSGSG–C–(CH_2_CONH_2_)
**MV HRC2 ^a^**	Ac–PPISLERLDVGTNLGNAIAKLEDAKELLESSDQILR–GSGSG–C–(PEG4–Chol)
**MV–HC**	Ac–PPISLERLDVGTNLGNAIAKLEDAKELLESSDQILR–GSGSG–C–(25HC)

^a^ Described in [54,57,73].

**Table 2:** Fusion inhibition activity, at 24 h, of MV HRC derived peptides.

Peptide	Fusion Inhibition	
	IC_50_ (μM)	IC_90_ (μM)
**MV HRC1 ^a^**	>10	>10
**MV HRC2 ^a^**	0.05 ± 0.01	~10
**MV–HC**	0.07 ± 0.005	~0.3

^a^ Data from [57].
